# Quality-of-life assessment in dementia: the use of DEMQOL and DEMQOL-Proxy total scores

**DOI:** 10.1007/s11136-016-1343-1

**Published:** 2016-06-18

**Authors:** Kia-Chong Chua, Anna Brown, Ryan Little, David Matthews, Liam Morton, Vanessa Loftus, Caroline Watchurst, Rhian Tait, Renee Romeo, Sube Banerjee

**Affiliations:** 1 Institute of Psychiatry, Psychology and Neuroscience, King’s College London, De Crespigny Park, London, SE5 8AF UK; 2 School of Psychology, University of Kent, Canterbury, UK; 3 Croydon Memory Service, South London and Maudsley NHS Foundation Trust, London, UK; 4 Kingston Integrated Health and Social Care Community Mental Health Team for Older People, South West London and St George’s NHS Trust, London, UK; 5 Brighton and Sussex Medical School, University of Sussex, Brighton, UK

**Keywords:** Dementia, Health-related quality-of-life, Exploratory factor analysis, Confirmatory factor analysis, Bifactor model

## Abstract

**Purpose:**

There is a need to determine whether health-related quality-of-life (HRQL) assessments in dementia capture what is important, to form a coherent basis for guiding research and clinical and policy decisions. This study investigated structural validity of HRQL assessments made using the DEMQOL system, with particular interest in studying domains that might be central to HRQL, and the external validity of these HRQL measurements.

**Methods:**

HRQL of people with dementia was evaluated by 868 self-reports (DEMQOL) and 909 proxy reports (DEMQOL-Proxy) at a community memory service. Exploratory and confirmatory factor analyses (EFA and CFA) were conducted using bifactor models to investigate domains that might be central to general HRQL. Reliability of the general and specific factors measured by the bifactor models was examined using omega (*ω*) and omega hierarchical (*ω*_h_) coefficients. Multiple-indicators multiple-causes models were used to explore the external validity of these HRQL measurements in terms of their associations with other clinical assessments.

**Results:**

Bifactor models showed adequate goodness of fit, supporting HRQL in dementia as a general construct that underlies a diverse range of health indicators. At the same time, additional factors were necessary to explain residual covariation of items within specific health domains identified from the literature. Based on these models, DEMQOL and DEMQOL-Proxy overall total scores showed excellent reliability (*ω*_h_ > 0.8). After accounting for common variance due to a general factor, subscale scores were less reliable (*ω*_h_ < 0.7) for informing on individual differences in specific HRQL domains. Depression was more strongly associated with general HRQL based on DEMQOL than on DEMQOL-Proxy (−0.55 vs −0.22). Cognitive impairment had no reliable association with general HRQL based on DEMQOL or DEMQOL-Proxy.

**Conclusions:**

The tenability of a bifactor model of HRQL in dementia suggests that it is possible to retain theoretical focus on the assessment of a general phenomenon, while exploring variation in specific HRQL domains for insights on what may lie at the ‘heart’ of HRQL for people with dementia. These data suggest that DEMQOL and DEMQOL-Proxy total scores are likely to be accurate measures of individual differences in HRQL, but that subscale scores should not be used. No specific domain was solely responsible for general HRQL at dementia diagnosis. Better HRQL was moderately associated with less depressive symptoms, but this was less apparent based on informant reports. HRQL was not associated with severity of cognitive impairment.

**Electronic supplementary material:**

The online version of this article (doi:10.1007/s11136-016-1343-1) contains supplementary material, which is available to authorized users.

## Introduction

In dementia, as in other long-term conditions, ‘adding life to years’ is as important as ‘adding years to life’ [1]. The objective of assessing health-related quality-of-life (HRQL) is to be able to measure this. While medications used for people with dementia target cognitive and psychiatric symptoms, these symptoms do not give a complete picture of how illness can affect daily life and life quality [2]. HRQL measures are designed to include a broad range of domains in which impairments can occur and also where function and enjoyment can be maintained or even improved despite the progressive nature of dementia [3]. The broad view afforded by HRQL assessment is of particular value in multifaceted conditions with a broad range of physical, psychological and social impacts, such as dementia, to ensure that overall treatment benefits or harms are not missed [4].

How to obtain meaningful measurement of HRQL in dementia is an area of active research. In a recent systematic review that compared psychometric properties of HRQL measures for Alzheimer’s disease and mixed dementia, the authors found 15 dementia-specific HRQL measures developed over the last 20 years [5]. The basis for measuring HRQL varies between instruments with different representations of what might be considered ‘good’ or ‘bad’ quality-of-life. There is a fundamental need to determine whether HRQL assessments in dementia capture what is important [6], to form a coherent basis for guiding research and clinical and policy decisions [7].

The first aim of this study was to explore structural validity of two HRQL measures, the DEMQOL and DEMQOL-Proxy, which relies on self- and informant report, respectively, for evaluating HRQL of people with dementia. These measures have shown good internal consistency, test–retest reliability and moderate evidence of validity in people with mild to moderate dementia for DEMQOL and mild, moderate and severe dementia for DEMQOL-Proxy [8]. Mulhern and colleagues [9] reported five domains in DEMQOL (cognition, negative emotion, positive emotion, social relationship and loneliness) and DEMQOL-Proxy (cognition, negative emotion, daily activities, positive emotion and appearance). In this study, we asked whether a coherent overall impression of a general phenomenon can emerge out of the complexities of multiple facets of HRQL. This entailed an investigation using unidimensional measurement models. Similar studies have also considered evidence of ‘essential unidimensionality’ [10] with a general factor in higher-order measurement models (e.g. second-order and bifactor models). These models retain substantive emphasis on a complex general phenomenon while recognising ‘construct-relevant multidimensionality’ in which multiple domain-specific factors are necessary to reflect the inherent content diversity of complex constructs [11–13]. A key benefit from such a focus is empirical clarity in how well every DEMQOL and DEMQOL-Proxy item discriminates individual differences in overall HRQL, though this is more apparent in bifactor models than in second-order models because items in the latter load indirectly on the general factor [13, 14]. Multidimensional measurement models without a general factor (i.e. first-order correlated-factors model) would not aid this investigation. We thus considered only unidimensional, second-order and bifactor models.

The second aim was to investigate what might be important for quality-of-life around the time of a dementia diagnosis and how this might be captured by subscale and/or overall total HRQL scores. This question is primarily informed by investigations of content and face validity [15, 16]. The items in DEMQOL and DEMQOL-Proxy were generated in a process that included focus group interviews to assure rigorous coverage of relevant issues from the perspectives of people with dementia and their carers [17]. However, certain HRQL domains could matter more than others at different stages of the illness experience. Two measurement models in particular allow for an examination of the domains most central to the HRQL concept—the second-order and the bifactor model. In second-order models, how well domain factors load on the second-order general factor provides an indication of the relative importance of domain-specific functioning for general HRQL. In bifactor models, the amount of variation in item responses explained by the general vs specific factor provides a similar indication. If individual differences in a particular domain were fully explained by the general factor, this might suggest that the domain lies ‘at heart’ of the HRQL concept [18]. In this study, the domains in DEMQOL and DEMQOL-Proxy were examined for such insights as well as their implications on scoring practices.

The third aim of this study was to examine external validity by investigating the clinical relevance of individual differences in HRQL in terms of how they co-vary with clinically important outcomes in dementia. For this purpose, multiple-indicators multiple-causes (MIMIC) models with latent variables were used so that these conclusions were not affected by measurement unreliability.

## Methods

### Sample

The study participants were community-dwelling individuals and their carers referred to the Croydon Memory Service, a service provided by the National Health Service (NHS) based in South London. This is a multidisciplinary and interagency team to generate early diagnosis in a timely manner, enabling choice and forward planning while people have capacity. It is designed to assess all incident cases in a given population. As well as diagnosis they provide information, and direct medical, psychological and social help to people with dementia and their family carers. They aim to prevent future crises by encouraging more effective and earlier help seeking and so reduce unwanted transition into care homes. The service model has been described in detail and has been subject to quantitative and qualitative evaluation [19, 20].

The subjects in this study were drawn from a series of consecutive cases who were referred to the service between December 2002 and June 2010. Cases were included in the analysis if, after a full multidisciplinary assessment (including physical examination, medical interview, laboratory and radiological investigations, neuropsychological assessment and mental state examination), they were given a formal clinical diagnosis of dementia using International Classification of Diseases (10th revision, ICD-10) diagnostic criteria [21]. They were excluded if they had not completed sufficient questions on the DEMQOL or DEMQOL-Proxy to allow the instruments to be scored. This sample therefore represents an analysis of routinely collected data of assessments of HRQL and other clinical assessments made at the time of first clinical diagnosis of dementia.

### Measures

DEMQOL (28 items) and DEMQOL-Proxy (31 items) are interviewer-administered measures which obtain self- and informant reports of the HRQL of people with dementia [17]. Items inquire about ‘feelings’, ‘memory’ and ‘everyday life’ of the person with dementia in the last week. A four-point Likert scale (1 = a lot, 2 = quite a bit, 3 = a little, 4 = not at all) is used to collect responses. Reverse scoring is required for five items in DEMQOL/DEMQOL-Proxy so that higher overall total scores reflect better HRQL.

The data routinely collected by the memory service included measures of clinical symptoms in dementia as well as the HRQL. These included clinical assessments of cognition, depression, neuropsychiatric symptoms and dependence in activities in daily living. The Mini-Mental State Examination [MMSE, 22] is a screening tool for general cognitive impairment, with higher overall total scores (range 0–30) indicating better performance, and studies have reported evidence of structural validity [23], predictive validity and reliability [24–26]. The 15-item Geriatric Depression Scale [GDS-15, 27] is a screening tool with higher overall total scores (range 0–15) indicating higher depression levels, and studies have reported evidence of concurrent validity [28–30] and diagnostic accuracy [30, 31]. The Neuropsychiatric Inventory [NPI, 32] is an assessment tool for frequency and severity of behavioural and psychological symptoms in dementia with higher overall scores (range 0–144) indicating poorer health, and studies have reported evidence of sensitivity to treatment-related changes [33, 34]. The Bristol Activities of Daily Living Scale [BADL, 35] is an assessment tool for functional decline among people with dementia in terms of their ability to carry out daily living activities independently with higher overall total scores (range 0–60) indicating more dependence, and studies have reported convergent validity and sensitivity to treatment-related changes [36, 37].

### Analysis

#### Exploratory factor analysis

To establish a framework for the psychological constructs involved in HRQL measured by the DEMQOL and DEMQOL-Proxy, we conducted exploratory factor analysis (EFA) with bifactor orthogonal rotation [11]. One to six latent factors were considered in the EFA to explore domain themes of individual differences in HRQL response patterns of self-report (DEMQOL) and informant report (DEMQOL-Proxy), respectively. Eigenvalues and model fit were considered to aid factor retention decisions.

#### Confirmatory factor analysis

Among HRQL domains previously reported in the DEMQOL and DEMQOL-Proxy literature [9], some were absent from the bifactor EFA models in this study. While EFA results constitute ‘absence of evidence’ of the domain presence, ‘evidence of absence’ is needed to confirm that the domain does not emerge as a specific factor in bifactor confirmatory factor analysis (CFA). Signs of such ‘factor collapse’ [14] include (a) small and non-statistically significant factor loadings on a specific domain; (b) non-statistically significant factor variance of a specific domain. Model estimation may also fail to converge since ‘factor collapse’ implies over-extraction (i.e. hypothesising too many factors).

After the initial CFA, three types of model comparisons were made: (1) we first compared bifactor CFA models with and without the domain factors that were absent in EFA. Relative to more complex models (e.g. more domain factors), models that offered more parsimonious explanations of the sample data (e.g. fewer domain factors) would show poorer exact model fit. If the relative decline in model fit was trivial, this result would add ‘evidence of absence’ to preceding investigations of factor collapse in the initial CFA; (2) having decided on a final bifactor CFA model for DEMQOL and DEMQOL-Proxy, respectively, we compared them with their nested second-order models. This alternative view of multidimensionality is a special case (i.e. nested model) of bifactor models [11, 38, 39], and thus second-order models can only fit the data worse. A recent simulation study has in fact demonstrated that the fit of bifactor model is unlikely to be challenged by second-order model and cautioned against relying on model comparison [40]; (3) we also included a comparison between bifactor CFA models and their strictly unidimensional counterparts to evaluate the extent in which the general HRQL factor was ‘essentially unidimensional’ [10] by comparing factor loadings on this general factor with those on the common factor of a strictly unidimensional model. These comparisons added to subsequent investigations aimed at informing whether individual differences in HRQL could be meaningfully interpreted with total scale scores and/or multiple subscale scores.

#### Reliability of model-based constructs

The CFA models imply ways in which DEMQOL (or DEMQOL-Proxy) scores could be used to reach conclusions about individual differences in HRQL. To see whether variation in overall total scores is mainly due to individual differences in general HRQL (i.e. good score reliability), we examined *factor saturation* using the omega hierarchical coefficient, *ω*_h_ [41, 42], which shows the percentage of variance in overall total scores that could be attributed to the target construct (general HRQL) in the presence of specific HRQL domains. As overall total scores have multiple sources of common variance (i.e. multidimensionality), reliability estimates would be more optimistic unless one of these sources of common variance is intended as the target construct using the *ω*_h_ [12]. We examined this issue using the omega (*ω*) coefficient, which shows the percentage of variance in overall total scores that could be attributed to all underlying factors (i.e. general *and* specific HRQL domains). Omega coefficients provide better estimates of measurement precision (reliability) than Cronbach’s alpha [13], which conveys similar information, but is a special case of omega appropriate only for unidimensional factor models indicated by items with approximately equal factor loadings [43]. By modifying the calculation of omega coefficients [12, 18], we also investigated reliability of subscales in the context of bifactor multidimensionality.

#### Clinical associations with HRQL individual differences

To investigate the external validity of model-based HRQL constructs, we estimated their correlations with clinically relevant outcomes. We added to the CFA models four observed clinical covariates: cognitive functioning (MMSE), depression (GDS), neuropsychiatric symptoms (NPI), and dependence in daily life activities (BADL). We also explored potential differences due to gender, and whether HRQL assessments were fully or partially complete (e.g. self-report available for fewer than all 28 DEMQOL items). By working with the latent constructs emerging from DEMQOL/DEMQOL-Proxy, the associations were not affected by unreliability in HRQL assessments.

#### Modelling

All analyses were conducted in Mplus version 7 [44]. With a four-point Likert scale, DEMQOL and DEMQOL-Proxy responses were most appropriately treated as order categorical data [45]. The analyses were hence based on polychoric correlations rather than Pearson’s correlations [46], and model parameters were estimated using the recommended diagonally weighted least squares (DWLS) estimator with robust standard errors, denoted ‘weighted least squares means and variance adjusted’ (WLSMV) in Mplus [47–49]. Overall model fit was evaluated in two ways. An *exact* fit between model predictions and observed data, within bounds of sampling error, would result in model Chi-square (*χ* ^2^) values that fail to reach statistical significance [50]. In addition to the Chi-square statistic, which is highly sensitive to sample size, a summary of *approximate* model fit was obtained. Approximate model fit is indicated by (1) low values of root mean square error of approximation [RMSEA, 51] where <0.10 is considered as acceptable and <0.05 as very good fit [52, 53]; and (2) high values of comparative fit index [CFI, 54] where >0.90 is considered as acceptable and >0.95 as very good fit [54, 55]. Modification indices, measured as improvement in exact model fit (or reduction in model *χ* ^2^ values) if constrained parameters are released, were used to inform modifications to the initial models. For models estimated with WLSMV, the DIFFTEST option in Mplus was required for model comparisons so as to obtain the correct Chi-square difference test (Δ*χ* ^2^) between models [44].

## Results

### Subjects

HRQL reports were obtained from 868 people with dementia and 909 informants. Details of the subjects with partially complete HRQL reports had slightly poorer health (e.g. GDS) than those for whom a full DEMQOL or DEMQOL-Proxy report was obtained (Table 1).

**Table 1:** Demographic and clinical characteristics of the study group by completeness of HRQL rating by self-report (DEMQOL) and informant report (DEMQOL-Proxy)

	DEMQOL		DEMQOL-Proxy	
	Complete	Partial	Complete	Partial
Participants	756	112	679	230
Age^a^	78.7 (8.5) *n* = 753	77.9 (8.2) *n* = 112	78.8 (8.1) *n* = 675	79.3 (9.0) *n* = 230
*Gender*				
Male	269	44	253	87
Female	487	68	426	143
*Ethnicity*				
White	657	86	580	191
Black	43	13	38	21
Asian	41	11	48	12
Unknown	15	2	13	6
*ICD-10* ^b^				
AD	425	54	369	119
AD mixed	192	33	175	67
Vascular	84	15	84	30
Others	15	5	22	5
Unknown	40	5	29	9
MMSE^a^ (scores: 0–30)	21.1 (5.1) *n* = 756	20.4 (5.3) *n* = 112	20.8 (5.3) *n* = 679	19.5 (5.6) *n* = 230
GDS^a^ (scores: 0–15)	3.0 (2.6) *n* = 692	3.1 (2.6) *n* = 105	2.9 (2.7) *n* = 619	3.2 (2.6) *n* = 198
NPI^a^ (scores: 0–144)	12.7 (13.0) *n* = 684	12.3 (14.1) *n* = 102	12.1 (12.1) *n* = 668	15.2 (16.9) *n* = 221
BADL^a^ (scores: 0–60)	9.5 (9.2) *n* = 691	10.3 (9.4) *n* = 105	9.5 (9.2) *n* = 671	11.6 (9.9) *n* = 225

^a^Mean (SD). Rate of missing data varies across variables; valid sample size (*n*) is reported

^b^ICD-10 diagnosis: Alzheimer’s disease, late/early onset (AD), Alzheimer’s disease, mixed type (AD mixed), vascular dementia (vascular), others/unspecified (others), ICD code not known (unknown)

As the Croydon Memory Service was set up to facilitate early diagnosis for community-dwelling older adults, study participants were a sample of people who were in early stages of illness. While cognitive impairment based on MMSE scores is consistent with this (Table 1), NPI scores on average were below the means reported in clinical trials for mild to moderate dementia [e.g. 33]. BADL scores of the present sample also showed less functional decline than those reported in the BADL tool development study [35] which had people with more severe cognitive impairment.

### EFA

With diverse outcomes in HRQL, a strictly unidimensional model was not tenable for DEMOQL (*χ* ^2^ = 4521.231 (*df* = 350), RMSEA = .117 (90 % CI .114–.120), CFI = .686) and DEMQOL-Proxy (*χ* ^2^ = 6235.656 (*df* = 434), RMSEA = .121 (90 % CI .119–.124), CFI = .681). Models with more domain factors gave better approximate fit even though model predictions did not reach an exact fit with DEMQOL and DEMQOL-Proxy data. Eigenvalues suggested a maximum of five factors might be considered for DEMQOL (10.540, 3.138, 1.690, 1.349, 1.187, 1.000) and a maximum of six factors for DEMQOL-Proxy (10.907, 3.277, 1.918, 1.581, 1.338, 1.207, 0.953). However, the ratio of the first two eigenvalues for DEMQOL (10.540 vs 3.138) and DEMQOL-Proxy (10.907 vs 3.277) suggested the presence of a strong general factor [13, 56].

For DEMQOL, we report the results of a bifactor EFA (**Model 1a**) that had a general HRQL factor and four domain-specific factors (supplementary Table 1). They were labelled as ‘positive emotion’ (POS: item 1, 3, 5, 6, 10), ‘negative emotion’ (NEG: item 4, 11, 12, 13), ‘loneliness’ (LON: item 8, 20) and ‘worries about cognition’ (COG: item 14, 15, 16, 17, 18, 19). Eleven DEMQOL items loaded saliently only on the general HRQL domain. For DEMQOL-Proxy, we report the results of a bifactor EFA (**Model 2a**) that had a general HRQL factor and five domain-specific factors (supplementary Table 2). They were labelled as ‘positive emotion’ (POS: item 1, 4, 6, 8, 11), ‘negative emotion’ (NEG: item 2, 3, 5, 7, 9, 10), ‘worries about appearance’ (APP: item 21, 22), ‘worries about finance-related tasks’ (FIN: item 23, 24, 25) and ‘worries about social relationships’ (SOC: item 27, 28, 29, 30). Eleven DEMQOL-Proxy items loaded saliently only on the general HRQL domain. Considerations that led to these final models included goodness of fit, interpretability of domain factor, fewer or weaker un-modelled cross-loadings and consistency with previous reports of multidimensionality [9, 17, 57].

Most of the HRQL domains reported in previous studies were replicated in the exploratory bifactor models of this study. However, the domain theme of ‘worries about social functioning’ (SOC) was absent from DEMQOL, whereas the domain theme of ‘worries about cognition’ (COG) was absent from DEMQOL-Proxy. These absent domains (SOC in DEMQOL and COG in DEMQOL-Proxy) formed the basis for investigating factor collapse in bifactor CFA models in the next stage.

### CFA

Based on published findings [9], an additional domain ‘worries about social relationships’ (SOC: item 21, 22, 23, 24, 25, 26) was hypothesised, giving five specific domains (POS, NEG, LON, COG and SOC) alongside a general HRQL domain for DEMQOL (**Model 1b**). Similarly, an additional domain ‘worries about cognition’ (COG: item 12, 13, 14, 15, 16, 17, 18, 19, 20) was hypothesised, giving six specific domains (POS, NEG, APP, FIN, SOC, COG) alongside a general HRQL domain for DEMQOL-Proxy (**Model 2b**). With adequate approximate fit, bifactor CFA models for DEMQOL (RMSEA = .062 (90 % CI .059–.065), CFI = .918) and DEMQOL-Proxy (RMSEA = .058 (90 % CI .055–.061), CFI = .932) did not show evidence of factor collapse. The SOC domain in DEMQOL (supplementary Table 3) and COG domain in DEMQOL-Proxy (supplementary Table 4) had statistically significant factor variances and factor loadings.

In these CFA models, most items loaded saliently (≥0.3) on the general factor. The specific factor loadings of items tended to be weaker than their general factor loadings. In other words, general HRQL explained more variance in the item responses than specific domains did. Items that indicated ‘positive emotion’ (POS) in DEMQOL and DEMQOL-Proxy were an exception. Their factor loadings showed statistically significant but relatively weaker contributions towards general HRQL.

To further investigate the presence of specific HRQL domains, DEMQOL Model 1b was formally compared with a nested bifactor model without a SOC domain (**Model 1c**). Similarly, DEMQOL-Proxy Model 2b was compared with a bifactor model without a COG domain (**Model 2c**). DIFFTEST results show the decline in model fit was statistically significant for DEMQOL Model 1c relative to Model 1b (Δ*χ* ^2^ = 172.023, *df* = 6) and DEMQOL-Proxy Model 2c relative to Model 2b (Δ*χ* ^2^ = 374.519, *df* = 9). The subsequent stage of investigation proceeded with Model 1b for DEMQOL and Model 2b for DEMQOL-Proxy.

Next, DEMQOL Model 1b and DEMQOL-Proxy Model 2b were compared with their nested second-order models. While the second-order models had acceptable approximate model fit for DEMQOL (RMSEA = .065 (90 % CI .062–.068), CFI = .904) and DEMQOL-Proxy (RMSEA = .066 (90 % CI .064–.069), CFI = .905), they showed a statistically significant decline in exact model fit relative to their bifactor model counterparts (DEMQOL: Δ*χ* ^2^ = 198.151, *df* = 18; DEMQOL-Proxy: Δ*χ* ^2^ = 369.875, *df* = 23). Given that model fit comparisons have ‘inherent statistical bias’ in favour of bifactor models [40], this result was not surprising and highlighted that modelling and scoring approaches should be based on model utility.

In the final round of model comparisons, DEMQOL Model 1b and DEMQOL-Proxy Model 2b were evaluated against their strictly unidimensional counterparts (supplementary Table 3 and 4, respectively). The unidimensional models had poor model fit due to content diversity [58], but their factor loadings served as a reference for evaluating the impact on general factor loadings when items also load on additional domain factors as in the bifactor model. For these items, their factor loadings on the general factor were smaller than their factor loadings in the unidimensional model. This parameter distortion (due to un-modelled complexity in the latter) was expected, but only five had a magnitude of 0.10 or larger in the 28-item DEMQOL (e.g. item 10: 0.24 vs 0.45) and 31-item DEMQOL-Proxy (e.g. item 14: 0.57 vs 0.78), respectively. The extent of these differences between the general factor and the unidimensional common factor lends support to the view that general HRQL is essentially unidimensional.

### Reliability

The general HRQL factor was a dominant influence on overall total scores in DEMQOL (supplementary Table 3: *ω*_h_ = 0.85) and DEMQOL-Proxy (supplementary Table 4: *ω*_h_ = 0.88). As there was more than one source of common variance underlying total scale scores (i.e. *GEN*, *POS*, *NEG*, *COG*, *LON*, *SOC* for DEMQOL; *GEN*, *POS* *NEG*, *APP*, *FIN*, *SOC*, *COG* for DEMQOL-Proxy), these would have led to more optimistic reliability estimates for DEMQOL (*ω* = 0.96) and DEMQOL-Proxy (*ω* = 0.96). Going by *ω* estimates, all DEMQOL and DEMQOL-Proxy subscales showed excellent reliability (*ω* > 0.80). When common variance in subscales was attributed to a general and specific source of influence, *ω*_h_ estimates showed that only 33–57 % of variation in subscale scores could be attributed to individual differences in specific HRQL domains. The POS domain was an exception. This subscale afforded excellent reliability in measuring individual differences in ‘positive emotion’ according to *ω* estimates and moderate reliability according to *ω*_h_ estimates in DEMQOL (*ω* = 0.86 vs *ω*_h_ = 0.65) and DEMQOL-Proxy (*ω* = 0.85 vs *ω*_h_ = 0.69).

### External validity

Six covariates (MMSE, GDS, NPI, BADL, gender and complete/partial HRQL assessment) were added to the DEMQOL bifactor CFA Model 1b, generating Model 1d. DEMQOL-Proxy Model 2b was augmented with an identical set of covariates, generating Model 2d. The associations between HRQL and clinical outcomes (adjusted for gender differences and whether HRQL data were complete/partial) are presented in Table 2.

**Table 2:** External validity of HRQL measurements (standardised coefficients)

Model 1d	HRQL	POS	NEG	COG	SOC	LON
DEMQOL (*n* = 724)						
Gender	.04	.03	−.03	−*.28*	−***.41***	***.51***
Ax	−.11	−.02	−.01	−.02	−.41	−.26
MMSE	.00	*.09*	−.08	.05	−.02	.10
NPI	−*.12*	.00	.01	*.19*	.01	−.07
GDS	−***.55***	−***.49***	−.***32***	−.01	*.28*	−.09
BADL	*.12*	−.08	−.07	*.18*	−.08	.07

Model 2d	HRQL	POS	NEG	COG	SOC	APP	FIN
DEMQOL-Proxy (n = 797)							
Gender	.***39***	−*.16*	.05	−.09	−.02	*.32*	−.14
Ax	−.02	.***32***	.23	.00	.01	−.01	.00
MMSE	−.07	.01	−.03	−.08	−.08	−.13	.00
NPI	−*.22*	−*.16*	−.***48***	−.05	.07	*.13*	−.01
GDS	−*.22*	−*.14*	−*.11*	.07	.08	.13	.13
BADL	−.01	−.***36***	.05	*.13*	−*.13*	−.***42***	−.11

Model 1d: *χ* ^2^ = 1339.318 (*df* = 460), RMSEA = .051 (90 % CI .048–.055), CFI = .903

Model 2d: *χ* ^2^ = 1599.858 (*df* = 550), RMSEA = .049 (90 % CI .046–.052), CFI = .931

Gender with female as reference group; Ax: fully complete HRQL assessments served as reference group for comparing with partially complete

*Standardised* coefficients are italicised, if *unstandardised* coefficients were statistically significant. *Standardised* coefficients are bolded if they exceed a magnitude of 0.30

Higher levels of self-reported general HRQL (DEMQOL) were moderately associated with less depression (GDS). When rated by informants, general HRQL (DEMQOL-Proxy) had only weak associations with clinical outcomes. Males tended to have better general HRQL according to their informants.

Higher levels of ‘positive emotion’ (POS) according to self-report (DEMQOL) were moderately associated with less depression (GDS). In informant report (DEMQOL-Proxy), higher levels of POS were moderately associated with less dependence in daily living (BADL). In self-report (DEMQOL), less ‘negative emotion’ (i.e. higher levels of NEG) was associated with less depression. In informant report, less ‘negative emotion’ was associated with more neuropsychiatric problems (NPI).

In self-report, associations between ‘worries about cognition’ (COG) and clinical outcomes were weak. Less worries (i.e. higher levels of COG) were associated with more neuropsychiatric problems (NPI) and dependence (BADL). For DEMQOL-Proxy, a weak association was found between less worries and more dependence (BADL).

In self-report, a weak association was found between less ‘worries about social relationship’ (i.e. higher levels of SOC) and more depression (GDS). Males also fared worse in this domain. In informant report, less worries showed a weak association with less dependence (BADL).

‘Loneliness’ (LON), a domain unique to DEMQOL, showed little association with clinical outcomes, only that males showed less worries (i.e. higher levels of LON). Less ‘worries about appearance’ (i.e. higher levels of APP), a domain unique to DEMQOL-Proxy, were moderately associated with more dependence (BADL). ‘Worries about finance-related tasks’ (FIN), also unique to DEMQOL-Proxy, showed little association with clinical outcomes.

## Discussion

### HRQL as a multidimensional phenomenon in dementia

HRQL is commonly articulated as a complex phenomenon that needs to be understood in terms of multiple health-related domains. The complex nature of HRQL in dementia is apparent from previous factor analytic studies [9] which have shed light on multiple themes of individual differences in item response patterns of DEMQOL and DEMQOL-Proxy. Using bifactor model perspectives, this paper confirms earlier findings that items covering a diverse range of health-related domains can be combined to an overall measure of HRQL in dementia. This finding aligns well with the substantive emphasis of HRQL assessments where the goal is to capture the overall balance of the impacts of diverse domains [59], particularly in treatment interventions that target broad outcomes [60]. By retaining strategic focus on general HRQL as the target construct, these analyses also show that some items (e.g. DEMQOL item 10) might be omitted from the assessment without affecting current levels of sensitivity in DEMQOL and DEMQOL-Proxy total scale scores to individual differences in a general complex phenomenon. This highlights the potential value of further analysis to consider the possibility of shorter versions of DEMQOL and DEMQOL-Proxy.

Furthermore, items from one domain, POS, had larger loadings on the domain factor than on the general factor, indicating that the POS-specific content was playing the more important role in responses to these items than the general HRQL factor. Reporting whether one had more ‘positive emotions’ or less ‘worries’ may also have different cognitive demands. Such influences have been reported in young children [61]. A recent population-based study has also reported an asymmetry of strong adverse reactions to deteriorations in health, alongside weak increases in well-being after health improvements [62]. Taken together, these issues may present challenges for overall HRQL scores to capture the variance of POS items, but this does not mean that positive emotion is not part of general HRQL. As POS items were the only items that required reverse-coding, the larger loadings on POS domain factor could also reflect this artefact [63–65]. Among studies investigating method effects [66–69], a multitrait–multimethod (MTMM) conceptual framework, comprising correlated-trait, correlated-uniqueness (CTCU) models, as well as correlated-trait, correlated-methods (CTCM) models, was employed to separate substantive content from method effects. While these analyses are beyond the scope of the present study, the orthogonality constraints of bifactor model framework provided the initial basis for speculating about the presence of potential method effects that are theoretically independent of individual differences in general HRQL [38]. However, these interpretations are post hoc, and thus preliminary, and a priori planned study designs that allow separating the substantive HRQL and common method effects (e.g. CTCU and CTCM models) are needed to reach a better understanding of this issue.

### What matters in HRQL in dementia?

The bifactor EFA models in this study suggest that ‘worries about social relationship’ might be a core influence on how people with dementia rate their HRQL using the 28-item DEMQOL, whereas ‘worries about cognition’ might be central to how informants rate HRQL of people with dementia using the 31-item DEMQOL-Proxy. However, direct investigation of factor collapse using bifactor CFA models and model comparisons did not support the conclusion that ‘worries about social relationship’ were at the ‘heart’ of self-report HRQL in dementia. These latter analyses also did not support the conclusion that ‘worries about cognition’ were at the ‘heart’ of informant-rated HRQL. Such potential differences between self-report and proxy-report HRQL warrant continued investigation in light of the body of literature showing that self- and informant perspectives are influenced by different things [70–73]. With respect to social relationships, Lawton [74] suggested that social behaviour in people with dementia is ‘a treatment goal that seems appropriate for an illness whose manifestations in general appear to represent estrangement from the external world’. As social functioning plays a pivotal role in the illness experience [75–78] as well as healthy ageing in general [79–82], factor collapse investigations using bifactor CFA such as those presented here may help shed light on whether social functioning could be considered a key clinical and policy focus when evaluating treatment interventions in dementia.

### Subscales and overall total scores

It has been argued that subscale scores should be calculated because HRQL by definition is a multidimensional concept and respective domain scores might help clarify treatment impact [5, 83]. However, the current study suggests that after controlling for general HRQL, subscales in DEMQOL and DEMQOL-Proxy explain little more and have poor score reliability, and therefore should not be used.

This conclusion should not obstruct efforts to understand the specific ways in which treatment interventions have an impact on HRQL. Overall total scores can demonstrate whether treatment interventions may or may not be effective at a global level, amidst ‘heterotypic continuity’ [84] in which evidence of ‘factor collapse’ can show how different domains of the same underlying phenomenon may be central at different stages of illness [85].

### Clinical associations with individual differences in HRQL

In line with prior research [4, 86], this study found that general HRQL had very little association with cognitive impairment and dependence in activities of daily living. Better HRQL was moderately associated with less depressive symptoms, but this was less apparent based on informant reports, possibly because depressive symptoms are less easily observed by informants [72].

It is worth noting that in development different items were found to work for self- and proxy report, so the 28 items in DEMQOL and 31 items in DEMQOL-Proxy are not identical. While this could have led to differences in construct validity of general HRQL, both measures do share four substantively similar domains (POS, NEG, COG, SOC). With content overlap in DEMQOL and DEMQOL-Proxy, there is also potential confusion over why some items that reflect negative emotion did not load on the NEG domain of DEMQOL, but they did load on NEG of DEMQOL-Proxy. In the context of bifactor models in which all domain factors are orthogonal, while negative emotions are integral elements of general HRQL, the elements of NEG domain carry ‘incremental prediction’ [87] which may reflect a form of negativity that is independent of self-report general HRQL. Following this logic, studies that have employed bifactor models have also shown that associations between these specific domains and external outcomes are not necessarily in the expected direction [e.g. 88, 89]. More definitive knowledge of the meaning of NEG (e.g. why it includes ‘frustrated’ and ‘irritable’ but not ‘sad’ and ‘distress’) and why NEG may differ in scope between self- and informant perspectives would require further research.

### Study limitations

First, this study focussed on individuals with dementia around the time of diagnosis and is predominantly a sample of mild to moderately severe dementia. At more advanced stages of illness, HRQL may change for self-report and/or informant perspectives. The association between general HRQL and clinical outcomes may also vary by illness severity. The data reported here may not therefore be generalisable to populations with severe dementia or possibly to those with more established dementia, in the years following diagnosis. Generalisability may be enhanced and selection bias minimised by the memory service being the setting for all diagnoses in a specific geographical area, as opposed to the subjects being drawn from a highly specialised tertiary referral service.

Second, this was a convenience sample and potential bias from missing data cannot be ruled out. However, all cases where there were data on HRQL were included and all the data were collected as part of routine baseline clinical assessment, so it is not likely that selection bias is a particular problem. Also difficulties in obtaining a full HRQL report (DEMQOL/DEMQOL-Proxy) were only weakly related to illness severity.

Third, metric invariance [90], or the absence of non-uniform differential item functioning (DIF), had not been examined prior to testing the MIMIC models. While MIMIC models aid the detection of uniform DIF, non-uniform DIF has to be investigated using multi-group factor analysis (MGFA). This presents two practical challenges for the current study: (1) with six covariates, more than 12 models (at least 6 for DEMQOL and DEMQOL-Proxy, respectively) have to be estimated for MGFA; and (2) with covariates such as MMSE, NPI, GDS and BADL, widely accepted cut-off scores are needed before conducting MGFA. In this study, we leveraged on the flexibility of MIMIC models for a concurrent investigation with multiple covariates that vary in nature of measurement (categories/scores). Furthermore, simulation studies have demonstrated that MIMIC model approaches compare favourably with established methods (e.g. MGFA) for investigating uniform DIF [91–93]. In this study, we detected some DIF effects (supplementary Table 5), but they did not affect conclusions about external validity (supplementary Table 6). Taken together, these MIMIC models serve as a useful first-stage investigation for generating hypotheses.

Finally, the themes that carry substantive relevance for HRQL in dementia may not be limited to the ones included in the DEMQOL measurement system. Given that other HRQL measures in dementia differ in content coverage, they may generate other findings about HRQL domains and what may matter at different stages of illness. DEMQOL is constrained by what is measurable on a Likert scale. Other measures and approaches may cover better other domains and determinants of what makes for quality-of-life in dementia, such as love or touch or time [94], which may be inaccessible to psychometrically based instruments.

## Electronic supplementary material

Below is the link to the electronic supplementary material.

Supplementary material 1 (DOC 420 kb)
