# Supplementary material for: Quality-of-life assessment in dementia: the use of DEMQOL and DEMQOL-Proxy total scores
Source: Qual Life Res. 2016 Jun 18;25(12):3107–18. doi: 10.1007/s11136-016-1343-1 (PMC5102947; doi:10.1007/s11136-016-1343-1)
Supplement: Supplementary file 1 — Supplementary material 1 (DOC 420 kb) [file 11136_2016_1343_MOESM1_ESM.doc]

**Tables (Online Supplement)**

Table 1 DEMQOL EFA Model 1a factor loadings (WLSMV, bi-Geomin orthogonal rotation)

| Qn | DEMQOL (n = 868) | **GEN** | *SE* | **Domain 1** | *SE* | **Domain 2** | *SE* | **Domain 3** | *SE* | **Domain 4** | *SE* |
| --- | --- | --- | --- | --- | --- | --- | --- | --- | --- | --- | --- |
| 1 | cheerful | .50 | *.04* | **.45** | *.03* | -.03 | *.03* | .16 | *.04* | -.08 | *.04* |
| 2 | worried or anxious | .64 | *.03* | .12 | *.03* | .15 | *.04* | -.03 | *.04* | .13 | *.04* |
| 3 | that you are enjoying life | .42 | *.04* | **.58** | *.03* | -.07 | *.03* | .20 | *.04* | -.10 | *.04* |
| 4 | frustrated | .53 | *.04* | .12 | *.04* | **.66** | *.05* | -.03 | *.03* | .07 | *.04* |
| 5 | confident | .41 | *.04* | **.51** | *.03* | .03 | *.04* | .04 | *.04* | .04 | *.04* |
| 6 | full of energy | .35 | *.05* | **.73** | *.03* | .06 | *.03* | -.08 | *.04* | -.07 | *.04* |
| 7 | sad | .62 | *.03* | .20 | *.04* | .18 | *.04* | .21 | *.04* | .07 | *.04* |
| 8 | lonely | .53 | *.04* | .00 | *.03* | .02 | *.03* | **.70** | *.04* | -.04 | *.04* |
| 9 | distressed | .71 | *.03* | .04 | *.03* | .18 | *.05* | .14 | *.05* | .23 | *.05* |
| 10 | lively | .26 | *.05* | **.77** | *.03* | .08 | *.04* | -.01 | *.03* | .00 | *.02* |
| 11 | irritable | .56 | *.03* | .00 | *.03* | **.39** | *.05* | -.01 | *.04* | .03 | *.04* |
| 12 | fed-up | .64 | *.03* | .21 | *.04* | **.40** | *.04* | .18 | *.04* | -.01 | *.03* |
| 13 | things that you wanted to do but couldn’t | .45 | *.03* | .15 | *.04* | **.29** | *.05* | -.06 | *.04* | -.02 | *.04* |
| 14 | forgetting things that happened recently | .59 | *.03* | -.07 | *.03* | .04 | *.03* | -.07 | *.03* | **.49** | *.04* |
| 15 | forgetting who people are | .55 | *.04* | -.23 | *.05* | -.01 | *.04* | .00 | *.04* | **.38** | *.05* |
| 16 | forgetting what day it is | .50 | *.04* | -.12 | *.04* | -.01 | *.04* | .02 | *.03* | **.44** | *.05* |
| 17 | your thoughts being muddled | .63 | *.03* | -.15 | *.04* | .03 | *.03* | .00 | *.03* | **.57** | *.04* |
| 18 | difficulty making decisions | .71 | *.03* | -.09 | *.04* | .01 | *.04* | -.02 | *.03* | **.31** | *.04* |
| 19 | poor concentration | .65 | *.03* | -.07 | *.04* | -.01 | *.03* | -.10 | *.04* | **.42** | *.04* |
| 20 | not having enough company | .54 | *.04* | .03 | *.03* | -.06 | *.04* | **.69** | *.05* | -.08 | *.05* |
| 21 | how you get on with people close to you | .70 | *.03* | -.27 | *.06* | .04 | *.04* | -.02 | *.04* | -.21 | *.06* |
| 22 | getting the affection that you want | .74 | *.03* | -.36 | *.06* | -.03 | *.03* | .12 | *.05* | -.36 | *.07* |
| 23 | people not listening to you | .70 | *.03* | -.32 | *.06* | -.06 | *.04* | -.01 | *.04* | -.05 | *.05* |
| 24 | making yourself understood | .64 | *.03* | -.26 | *.05* | -.02 | *.04* | -.05 | *.04* | .12 | *.05* |
| 25 | getting help when you need it | .74 | *.03* | -.25 | *.06* | -.08 | *.05* | -.02 | *.04* | .03 | *.05* |
| 26 | getting to the toilet in time | .56 | *.04* | -.20 | *.06* | -.05 | *.06* | -.15 | *.06* | -.04 | *.05* |
| 27 | how you feel in yourself | .78 | *.02* | .04 | *.04* | -.21 | *.05* | -.23 | *.05* | .02 | *.03* |
| 28 | your health overall | .64 | *.03* | .18 | *.05* | -.12 | *.05* | -.32 | *.05* | -.05 | *.04* |

χ^2^ = 738.844 (df = 248), RMSEA = .048 (90% CI = .044 - .052), CFI = .963

Factor loadings in black print are statistically significant, those in grey are not. Loadings are in **bold** to clarify item assignment for each domain factor. Provisional labels for GEN: general HRQL, Domain 1: positive emotion, Domain 2: negative emotion, Domain 3: loneliness, Domain 4: worries about cognition.

Table 2 DEMQOL-Proxy EFA Model 2a factor loadings (WLSMV with bi-Geomin orthogonal rotation)

| Qn | DEMQOL-Proxy (n = 909) | **GEN** | *SE* | **Domain 1** | *SE* | **Domain 2** | *SE* | **Domain 3** | *SE* | **Domain 4** | *SE* | **Domain 5** | *SE* |
| --- | --- | --- | --- | --- | --- | --- | --- | --- | --- | --- | --- | --- | --- |
| 1 | cheerful | .26 | *.04* | **.50** | *.03* | .44 | *.04* | -.05 | *.04* | .03 | *.03* | -.04 | *.03* |
| 2 | worried or anxious | .50 | *.03* | .01 | *.03* | **.53** | *.03* | -.10 | *.04* | .02 | *.04* | -.01 | *.03* |
| 3 | frustrated | .51 | *.03* | .09 | *.03* | **.47** | *.03* | .04 | *.04* | -.08 | *.04* | -.03 | *.04* |
| 4 | full of energy | .19 | *.05* | **.86** | *.03* | .02 | *.03* | .03 | *.03* | -.06 | *.04* | .07 | *.04* |
| 5 | sad | .50 | *.03* | .09 | *.03* | **.62** | *.03* | -.05 | *.04* | .01 | *.04* | .04 | *.03* |
| 6 | content | .30 | *.04* | **.41** | *.03* | .50 | *.04* | -.02 | *.03* | .07 | *.04* | .10 | *.04* |
| 7 | distressed | .57 | *.03* | -.02 | *.03* | **.56** | *.03* | .01 | *.04* | -.05 | *.04* | -.02 | *.03* |
| 8 | lively | .19 | *.04* | **.84** | *.03* | .12 | *.04* | .01 | *.03* | -.05 | *.04* | .00 | *.03* |
| 9 | irritable | .35 | *.04* | .05 | *.03* | **.50** | *.03* | .16 | *.04* | -.08 | *.05* | -.01 | *.04* |
| 10 | fed-up | .55 | *.03* | .15 | *.03* | **.55** | *.03* | .10 | *.04* | -.02 | *.03* | .06 | *.04* |
| 11 | that he/she has things to look forward to | .19 | *.04* | **.51** | *.03* | .23 | *.04* | .11 | *.04* | .08 | *.05* | -.07 | *.04* |
| 12 | his/her memory in general | .67 | *.03* | -.20 | *.05* | -.01 | *.02* | -.37 | *.05* | -.18 | *.07* | -.01 | *.02* |
| 13 | forget things that happened a long time ago | .58 | *.03* | -.09 | *.05* | -.01 | *.04* | -.10 | *.05* | -.01 | *.05* | -.10 | *.05* |
| 14 | forgetting things that happened recently | .81 | *.02* | -.20 | *.05* | -.07 | *.03* | -.35 | *.05* | -.18 | *.07* | -.01 | *.02* |
| 15 | forgetting people’s names | .69 | *.02* | -.17 | *.04* | -.13 | *.04* | -.06 | *.05* | -.11 | *.06* | -.09 | *.04* |
| 16 | forgetting where he/she is | .61 | *.03* | -.03 | *.04* | .13 | *.05* | .04 | *.04* | .09 | *.05* | -.20 | *.06* |
| 17 | forgetting what day it is | .74 | *.02* | -.04 | *.04* | -.05 | *.03* | -.06 | *.04* | .03 | *.05* | -.14 | *.04* |
| 18 | his/her thoughts being muddled | .83 | *.02* | -.03 | *.03* | .00 | *.03* | -.03 | *.04* | .00 | *.04* | -.13 | *.04* |
| 19 | difficulty making decisions | .78 | *.02* | .00 | *.03* | -.04 | *.03* | -.08 | *.04* | .10 | *.04* | -.05 | *.04* |
| 20 | making him/herself understood | .70 | *.03* | -.03 | *.04* | -.04 | *.04* | .03 | *.04* | .02 | *.04* | -.10 | *.05* |
| 21 | keeping him/herself clean | .59 | *.05* | .03 | *.04* | .00 | *.04* | **.72** | *.07* | -.05 | *.05* | .06 | *.05* |
| 22 | keeping him/herself looking nice | .58 | *.04* | -.01 | *.04* | -.01 | *.03* | **.62** | *.06* | .01 | *.04* | .09 | *.05* |
| 23 | getting what he/she wants from the shops | .60 | *.04* | -.02 | *.04* | -.05 | *.04* | .19 | *.05* | **.45** | *.05* | .11 | *.05* |
| 24 | using money to pay for things | .60 | *.05* | .02 | *.03* | -.01 | *.03* | -.02 | *.04* | **.69** | *.05* | -.09 | *.05* |
| 25 | looking after his/her finances | .56 | *.04* | .02 | *.03* | .05 | *.04* | -.09 | *.05* | **.60** | *.05* | -.01 | *.03* |
| 26 | things taking longer than they used to | .63 | *.03* | .06 | *.04* | -.06 | *.04* | -.09 | *.04* | .12 | *.05* | .23 | *.04* |
| 27 | getting in touch with people | .62 | *.04* | -.06 | *.04* | -.03 | *.04* | .08 | *.05* | .25 | *.05* | **.37** | *.05* |
| 28 | not having enough company | .55 | *.03* | .03 | *.04* | .16 | *.04* | .10 | *.05* | .19 | *.05* | **.29** | *.05* |
| 29 | not being able to help other people | .49 | *.04* | .00 | *.03* | .00 | *.03* | .08 | *.04* | -.02 | *.03* | **.73** | *.04* |
| 30 | not playing a useful part in things | .53 | *.04* | .03 | *.03* | .10 | *.04* | .03 | *.03* | -.03 | *.03* | **.58** | *.04* |
| 31 | his/her physical health | .44 | *.03* | .03 | *.04* | .10 | *.04* | -.01 | *.05* | .04 | *.05* | .22 | *.04* |

χ^2^ = 754.149 (df = 294), RMSEA = .041 (90% CI = .038 - .045), CFI = .975

Factor loadings in black print are statistically significant, those in grey are not. Loadings are in **bold** to clarify item assignment for each domain factor. Provisional labels for GEN: general HRQL, Domain 1: positive emotion, Domain 2: negative emotion, Domain 3: worries about appearance, Domain 4: worries about daily financial tasks, Domain 5: worries about social relationships.

Table 3 DEMQOL bifactor CFA (Model 1b) and a strictly unidimensional model (UNI-D)

| Qn | h^2^ | **GEN HRQL** | *SE* | **POS** | *SE* | **NEG** | *SE* | **COG** | *SE* | **LON** | *SE* | **SOC** | *SE* | **UNI-D** | *SE* |
| --- | --- | --- | --- | --- | --- | --- | --- | --- | --- | --- | --- | --- | --- | --- | --- |
| 1 | .48 | .47 | *.03* | .51 | *.03* |  |  |  |  |  |  |  |  | .53 | *.03* |
| 2 | .48 | .70 | *.02* |  |  |  |  |  |  |  |  |  |  | .65 | *.02* |
| 3 | .52 | .38 | *.03* | .61 | *.03* |  |  |  |  |  |  |  |  | .48 | *.03* |
| 4 | .91 | .57 | *.03* |  |  | .77 | *.08* |  |  |  |  |  |  | .61 | *.03* |
| 5 | .44 | .40 | *.03* | .53 | *.03* |  |  |  |  |  |  |  |  | .48 | *.03* |
| 6 | .66 | .31 | *.03* | .75 | *.02* |  |  |  |  |  |  |  |  | .49 | *.03* |
| 7 | .48 | .70 | *.03* |  |  |  |  |  |  |  |  |  |  | .66 | *.02* |
| 8 | .80 | .54 | *.03* |  |  |  |  |  |  | .71 | *.02* |  |  | .62 | *.03* |
| 9 | .63 | .79 | *.03* |  |  |  |  |  |  |  |  |  |  | .74 | *.03* |
| 10 | .66 | .24 | *.04* | .77 | *.02* |  |  |  |  |  |  |  |  | .45 | *.03* |
| 11 | .43 | .59 | *.03* |  |  | .29 | *.05* |  |  |  |  |  |  | .59 | *.03* |
| 12 | .58 | .69 | *.02* |  |  | .32 | *.05* |  |  |  |  |  |  | .70 | *.02* |
| 13 | .30 | .47 | *.03* |  |  | .28 | *.05* |  |  |  |  |  |  | .48 | *.03* |
| 14 | .60 | .58 | *.03* |  |  |  |  | .51 | *.04* |  |  |  |  | .66 | *.02* |
| 15 | .49 | .52 | *.04* |  |  |  |  | .47 | *.04* |  |  |  |  | .58 | *.03* |
| 16 | .47 | .48 | *.04* |  |  |  |  | .49 | *.04* |  |  |  |  | .56 | *.03* |
| 17 | .74 | .62 | *.03* |  |  |  |  | .60 | *.04* |  |  |  |  | .72 | *.02* |
| 18 | .61 | .72 | *.03* |  |  |  |  | .32 | *.04* |  |  |  |  | .74 | *.02* |
| 19 | .61 | .64 | *.03* |  |  |  |  | .45 | *.03* |  |  |  |  | .70 | *.02* |
| 20 | .79 | .53 | *.04* |  |  |  |  |  |  | .71 | *.02* |  |  | .63 | *.03* |
| 21 | .61 | .58 | *.04* |  |  |  |  |  |  |  |  | .53 | *.05* | .65 | *.03* |
| 22 | .76 | .57 | *.04* |  |  |  |  |  |  |  |  | .66 | *.05* | .66 | *.03* |
| 23 | .64 | .60 | *.04* |  |  |  |  |  |  |  |  | .53 | *.04* | .66 | *.03* |
| 24 | .48 | .60 | *.04* |  |  |  |  |  |  |  |  | .35 | *.05* | .61 | *.03* |
| 25 | .61 | .67 | *.04* |  |  |  |  |  |  |  |  | .39 | *.05* | .69 | *.03* |
| 26 | .36 | .48 | *.05* |  |  |  |  |  |  |  |  | .36 | *.06* | .50 | *.04* |
| 27 | .58 | .76 | *.02* |  |  |  |  |  |  |  |  |  |  | .71 | *.02* |
| 28 | .40 | .63 | *.03* |  |  |  |  |  |  |  |  |  |  | .59 | *.03* |
|  | ω | .96 |  | .86 |  | .82 |  | .89 |  | .88 |  | .89 |  | .94 |  |
|  | ω*_h_* | .85 |  | .65 |  | .28 |  | .35 |  | .57 |  | .35 |  | .94 |  |

χ^2^ = 1420.583 (df = 328), RMSEA = .062 (90% CI = .059 - .065), CFI = .918

h^2^: communalities in bifactor model; SE: standard error; ω: Omega coefficient; ω*_h_*: Omega hierarchical coefficient

Table 4 DEMQOL-Proxy bifactor CFA (Model 2b) and a strictly unidimensional model (UNI-D)

| Qn | h^2^ | **GEN HRQL** | *SE* | **POS** | *SE* | **NEG** | *SE* | **APP** | *SE* | **FIN** | *SE* | **SOC** | *SE* | **COG** | *SE* | **UNI-D** | *SE* |
| --- | --- | --- | --- | --- | --- | --- | --- | --- | --- | --- | --- | --- | --- | --- | --- | --- | --- |
| 1 | .45 | .39 | *.03* | .55 | *.03* |  |  |  |  |  |  |  |  |  |  | .44 | *.03* |
| 2 | .51 | .57 | *.03* |  |  | .43 | *.04* |  |  |  |  |  |  |  |  | .62 | *.02* |
| 3 | .51 | .56 | *.03* |  |  | .44 | *.04* |  |  |  |  |  |  |  |  | .61 | *.02* |
| 4 | .70 | .19 | *.04* | .82 | *.02* |  |  |  |  |  |  |  |  |  |  | .39 | *.03* |
| 5 | .60 | .61 | *.03* |  |  | .48 | *.04* |  |  |  |  |  |  |  |  | .67 | *.02* |
| 6 | .45 | .48 | *.03* | .47 | *.03* |  |  |  |  |  |  |  |  |  |  | .49 | *.03* |
| 7 | .65 | .64 | *.03* |  |  | .49 | *.04* |  |  |  |  |  |  |  |  | .69 | *.02* |
| 8 | .78 | .21 | *.04* | .86 | *.02* |  |  |  |  |  |  |  |  |  |  | .41 | *.03* |
| 9 | .41 | .40 | *.04* |  |  | .50 | *.04* |  |  |  |  |  |  |  |  | .47 | *.03* |
| 10 | .63 | .66 | *.03* |  |  | .44 | *.04* |  |  |  |  |  |  |  |  | .70 | *.02* |
| 11 | .35 | .26 | *.04* | .53 | *.03* |  |  |  |  |  |  |  |  |  |  | .32 | *.03* |
| 12 | .62 | .47 | *.03* |  |  |  |  |  |  |  |  |  |  | .63 | *.03* | .67 | *.02* |
| 13 | .37 | .46 | *.04* |  |  |  |  |  |  |  |  |  |  | .40 | *.04* | .53 | *.03* |
| 14 | .86 | .57 | *.03* |  |  |  |  |  |  |  |  |  |  | .73 | *.03* | .78 | *.02* |
| 15 | .51 | .49 | *.03* |  |  |  |  |  |  |  |  |  |  | .52 | *.03* | .61 | *.02* |
| 16 | .38 | .57 | *.04* |  |  |  |  |  |  |  |  |  |  | .24 | *.05* | .59 | *.03* |
| 17 | .55 | .61 | *.03* |  |  |  |  |  |  |  |  |  |  | .42 | *.03* | .69 | *.02* |
| 18 | .68 | .72 | *.02* |  |  |  |  |  |  |  |  |  |  | .40 | *.03* | .78 | *.02* |
| 19 | .61 | .70 | *.02* |  |  |  |  |  |  |  |  |  |  | .36 | *.03* | .74 | *.02* |
| 20 | .47 | .61 | *.03* |  |  |  |  |  |  |  |  |  |  | .33 | *.04* | .64 | *.03* |
| 21 | .82 | .56 | *.05* |  |  |  |  | .71 | *.03* |  |  |  |  |  |  | .63 | *.04* |
| 22 | .82 | .56 | *.04* |  |  |  |  | .71 | *.03* |  |  |  |  |  |  | .61 | *.03* |
| 23 | .54 | .63 | *.04* |  |  |  |  |  |  | .38 | *.05* |  |  |  |  | .62 | *.03* |
| 24 | .97 | .61 | *.04* |  |  |  |  |  |  | .77 | *.07* |  |  |  |  | .65 | *.03* |
| 25 | .62 | .60 | *.03* |  |  |  |  |  |  | .50 | *.05* |  |  |  |  | .62 | *.03* |
| 26 | .45 | .67 | *.03* |  |  |  |  |  |  |  |  |  |  |  |  | .61 | *.02* |
| 27 | .54 | .66 | *.03* |  |  |  |  |  |  |  |  | .32 | *.05* |  |  | .63 | *.03* |
| 28 | .44 | .62 | *.03* |  |  |  |  |  |  |  |  | .22 | *.05* |  |  | .58 | *.03* |
| 29 | .87 | .49 | *.04* |  |  |  |  |  |  |  |  | .79 | *.07* |  |  | .55 | *.03* |
| 30 | .61 | .57 | *.03* |  |  |  |  |  |  |  |  | .53 | *.06* |  |  | .59 | *.03* |
| 31 | .25 | .50 | *.03* |  |  |  |  |  |  |  |  |  |  |  |  | .45 | *.03* |
|  | ω | .96 |  | .85 |  | .88 |  | .90 |  | .88 |  | .85 |  | .92 |  | .94 |  |
|  | ω*_h_* | .88 |  | .69 |  | .35 |  | .56 |  | .39 |  | .33 |  | .34 |  | .94 |  |

χ^2^ = 1647.018 (df = 406), RMSEA = .058 (90% CI = .055 - .061), CFI = .932

h^2^: communalities in bifactor model; SE: standard error; ω: Omega coefficient; ω*_h_*: Omega hierarchical coefficient

Table 5 Differential item functioning (DIF) effects (standardised coefficients)

| **Model 1d** | DEMQOL | Gender | MMSE | NPI | GDS | BADL |
| --- | --- | --- | --- | --- | --- | --- |
| Item 5 | *confident* |  |  |  | .17 |  |
|  |  |  |  |  |  |  |
| **Model 2d** | DEMQOL-Proxy | Gender | MMSE | NPI | GDS | BADL |
| Item 6 | *content* |  |  | -.16 |  | .16 |
| Item 9 | *irritable* |  |  | -.22 |  |  |
| Item 20 | *making self understood* | **- .34** | .19 |  |  |  |
| Item 28 | *not having enough company* | **.58** |  |  |  |  |
|  |  |  |  |  |  |  |

Table 6 External validity of HRQL measurements adjusted for DIF (Compare with Table 2).

| **DEMQOL (n=724)** | | | | | | | |
| --- | --- | --- | --- | --- | --- | --- | --- |
| **Model 1d** | HRQL | POS | NEG | COG | SOC | LON |  |
| Gender | .05 | .03 | -.04 | -.30 | **-.43** | **.50** |  |
| Ax | -.12 | -.02 | .01 | .00 | -.38 | -.25 |  |
| MMSE | .01 | .09 | -.08 | .04 | -.02 | .10 |  |
| NPI | -.13 | .00 | .02 | .21 | .03 | -.06 |  |
| GDS | **-.58** | **-.52** | -.29 | .05 | **.34** | -.07 |  |
| BADL | .12 | -.08 | -.08 | .17 | -.10 | .07 |  |
|  | | | | | | | |
| **DEMQOL-Proxy (n=797)** | | | | | | | |
| **Model 2d** | HRQL | POS | NEG | COG | SOC | APP | FIN |
| Gender | .30 | -.13 | .16 | .07 | -.04 | **.40** | -.05 |
| Ax | -.02 | **.32** | .24 | .00 | .02 | -.01 | .00 |
| MMSE | -.09 | .01 | -.01 | -.08 | -.06 | -.11 | .03 |
| NPI | -.23 | -.14 | **-.41** | -.04 | .08 | .14 | .00 |
| GDS | -.21 | -.14 | -.12 | .07 | .08 | .12 | .12 |
| BADL | -.05 | **-.38** | .09 | .17 | -.09 | **-.39** | -.07 |

Model 1d: χ^2^ = 1224.766 (df = 458), RMSEA = .048 (90% CI = .045 - .051), CFI = .915

Model 2d: χ^2^ = 1505.880 (df = 544), RMSEA = .047 (90% CI = .044 - .050), CFI = .937

Gender with female as reference group; Ax: Fully complete HRQL assessments served as reference group for comparing with partially complete.

*Standardised* coefficients are shaded in gray only if *unstandardised* coefficients were statistically significant. *Standardised* coefficients are bolded if they exceed a magnitude of 0.30.
